# Increased plasma lipids in triple-negative breast cancer and impairment in HDL functionality in advanced stages of tumors

**DOI:** 10.1038/s41598-023-35764-7

**Published:** 2023-06-02

**Authors:** Maria Isabela Bloise Alves Caldas Sawada, Monique de Fátima Mello Santana, Mozania Reis, Sayonara Ivana Santos de Assis, Lucas Alves Pereira, Danielle Ribeiro Santos, Valéria Sutti Nunes, Maria Lucia Cardillo Correa-Giannella, Luiz Henrique Gebrim, Marisa Passarelli

**Affiliations:** 1 Programa de Pós-Graduação em Medicina, Universidade Nove de Julho (UNINOVE), São Paulo, Brazil; 2 Centro de Referência da Saúde da Mulher (Hospital Pérola Byington), São Paulo, Brazil; 3 Hospital da Força Aérea de São Paulo, São Paulo, Brazil; 4 Laboratório de Lípides (LIM10), Hospital das Clínicas (HCFMUSP) da Faculdade de Medicina da Universidade de São Paulo, São Paulo, Brazil; 5 Unidade Básica de Saúde Dra. Ilza Weltman Hutzler, São Paulo, Brazil; 6 Laboratório de Carboidratos e Radioimunoensaio Lípides (LIM18), Hospital das Clínicas (HCFMUSP) da Faculdade de Medicina da Universidade de São Paulo, São Paulo, Brazil

**Keywords:** Breast cancer, Cancer metabolism, Prognostic markers, Cancer, Breast cancer, Cancer metabolism, Cancer, Biomarkers, Oncology

## Abstract

The association between plasma lipids and breast cancer (BC) has been extensively explored although results are still conflicting especially regarding the relationship with high-density lipoprotein cholesterol (HDLc) levels. HDL mediates cholesterol and oxysterol removal from cells limiting sterols necessary for tumor growth, inflammation, and metastasis and this may not be reflected by measuring HDLc. We addressed recently diagnosed, treatment-naïve BC women (n = 163), classified according to molecular types of tumors and clinical stages of the disease, in comparison to control women (CTR; n = 150) regarding plasma lipids and lipoproteins, HDL functionality and composition in lipids, oxysterols, and apo A-I. HDL was isolated by plasma discontinuous density gradient ultracentrifugation. Lipids (total cholesterol, TC; triglycerides, TG; and phospholipids, PL) were determined by enzymatic assays, apo A-I by immunoturbidimetry, and oxysterols (27, 25, and 24-hydroxycholesterol), by gas chromatography coupled with mass spectrometry. HDL-mediated cell cholesterol removal was determined in macrophages previously overloaded with cholesterol and ^14^C-cholesterol. Lipid profile was similar between CTR and BC groups after adjustment per age. In the BC group, lower concentrations of TC (84%), TG (93%), PL (89%), and 27-hydroxicholesterol (61%) were observed in HDL, although the lipoprotein ability in removing cell cholesterol was similar to HDL from CRT. Triple-negative (TN) BC cases presented higher levels of TC, TG, apoB, and non-HDLc when compared to other molecular types. Impaired HDL functionality was observed in more advanced BC cases (stages III and IV), as cholesterol efflux was around 28% lower as compared to stages I and II. The altered lipid profile in TN cases may contribute to channeling lipids to tumor development in a hystotype with a more aggressive clinical history. Moreover, findings reinforce the dissociation between plasma levels of HDLc and HDL functionality in determining BC outcomes.

## Introduction

Female breast cancer (BC) is the most commonly diagnosed cancer worldwide, accounting for 11.7% of all cancer cases, and is the fifth leading cause of cancer-related deaths, representing 6.9% of all cancer deaths^1^. Breast cancer is considered a heterogeneous disease, and its molecular classification is widely used to determine treatment options and prognosis. This classification is based on the expression of estrogen and progesterone receptors (luminal A; LA, and luminal B; LB), human epidermal growth factor receptor 2 (HER2), or the absence of these receptors (triple-negative; TN)^2,3^.

In recent decades, evidence has emerged linking plasma lipid levels to the development and  worse outcomes of BC^4–7^. This is related to tumor cell reprogramming that enables greater lipid uptake for cell division^8^. However, clinical and epidemiological data have shown mixed results, with some studies suggesting a positive or negative impact of hyperlipidemia on BC incidence, while others suggest no impact^9–13^. Similarly, the association between statins and the risk of BC is still controversial^14,15^.

Much attention has been focused on the role of cholesterol in high-density lipoproteins (HDLc) in relation to BC risk and prevention, with most studies suggesting a protective role of plasma HDLc^4,10,11^. These results may be due to the beneficial activities of HDL in removing excess cholesterol and oxysterols from tumor cells^16^. Furthermore, HDL has antioxidant and anti-inflammatory activities and acts as a carrier for bioactive lipids, proteins, and microRNAs that may modulate tumor growth and progression^17,18^. However, there is still controversy surrounding this topic, with some studies showing only a weak association between HDLc and BC, while others show a positive or no association at all^9,12,13,19^.

Similar to the role of HDL in atherosclerosis and other chronic non-communicable diseases, it is possible that the HDLc metric may not be the best predictor variable for determining BC incidence and progression^20^. Therefore, alterations in HDL functionality should be considered, particularly when taking into account the modulation of this lipoprotein's composition and function by the tumor microenvironment^21^.

HDL is well known for its ability to remove cellular lipids, allowing cholesterol for uptake by the liver, secretion into bile, and excretion in feces through reverse cholesterol transport. Similarly, in the case of tumor cells, reverse cholesterol transport can be considered a defensive mechanism that prevents cholesterol accumulation that supports cell proliferation and metastasis. The expression of HDL receptors, such as ATP-binding cassette transporter A-1 (ABCA-1) and scavenger receptor class B type 1 (SR-B1), that mediated cholesterol exportation is altered in tumors and is related to epithelial-mesenchymal transition, tumor growth, and metastasis^22–25^. HDL also mediates the transport of oxysterols produced intracellularly by the oxidation of the cholesterol molecule. Due to its higher hydrophobicity compared to other oxysterols and cholesterol, 27 hydroxycholesterol (27HC) can easily diffuse across the plasma membrane^26^. Additionally, its transfer to HDL is facilitated by the ATP-binding cassette transporters G-1 (ABCG-1) located in the cell membrane of tumor cells and macrophages that infiltrate the tumor area^27^. Then, 27HC flux outside cells is considered an additional route for reverse cholesterol transport, limiting sterol content that is associated with cell inflammation, oxidative stress, and proliferation^18,28^. 27 hydroxycholesterol also acts as a selective estrogen receptor modulator (SERM), promoting tumor growth and metastasis in BC. In human BC samples, the expression of the enzymes involved in the production of 27HC (CYP27A1) and metabolism (CYP7B1) is respectively increased and decreased and is associated with poor tumor prognosis^29^. Furthermore, tumor metastasis is related to 27HC-dependent activation of the liver X receptor (LXR)^29–32^.

HDL removes cholesterol and oxysterols from cells, making acceptable the idea that it limits the intracellular accumulation of sterols and their negative impact on BC. In this way, it was addressed in newly diagnosed women with BC categorized according to the clinical stage of the disease and molecular classification of the tumor, without pharmacological or surgical intervention, compared to control women (CTR): (1) plasma lipid profile [total cholesterol (TC), HDLc, apolipoprotein B (apoB) and triglycerides (TG)]; (2) the composition of isolated HDL in lipids, main species of oxysterols and apolipoprotein A-I (apoA-I); and (3) the ability of HDL to mediate cholesterol removal from macrophages.

Plasma lipids were similar between controls (CTR) and all women with BC, but when categorized according to tumor molecular type, triple-negative (TN) BC cases had higher plasma TC, TG, and apoB values compared to other molecular types. Despite having lower levels of TC, phospholipids (PL), and 27HC, HDL from women with BC maintained its ability to remove cellular cholesterol when compared to HDL from CTR. In advanced BC (covering clinical stages III and IV), despite similar composition in apoA-I and lipids, HDL had an impaired ability to remove cholesterol from macrophages.

## Material and methods

Two hundred and one women newly diagnosed with BC between 18 and 80 years old in any clinical stage and with the molecular classification of the tumor were recruited at Hospital Pérola Byington. A hundred and fifty-seven women without any type of cancer were recruited at Universidade de São Paulo and at Unidade Básica de Saúde Dra. Ilza Weltman Hutzler (control group; CTR). Exclusion criteria were diabetes mellitus, chronic kidney disease (estimated glomerular filtration rate < 60 mL/min/1.73m2), autoimmune diseases, smokers, alcoholics, use of contraceptives, and hormone replacement therapy, pregnancy, previous history of any cancer, and in situ breast disease. After excluding those who did not meet the eligibility criteria, 150 CTR and 163 women with BC remained in the study. All participants were informed about the study and signed an informed written consent previously approved by institutional Ethics Committees, in accordance with the Declaration of Helsinki, including approval for publication (Universidade Nove de Julho, # 3.139.460; Centro de Referência da Saúde da Mulher, Hospital Pérola Byington, #3.225.220; and Hospital das Clínicas da Faculdade de Medicina da Universidade de São Paulo, #3.317.909).

The molecular classification of tumors was obtained from medical records being accessed immunohistochemically after percutaneous biopsy, according to the American College of Pathologists^33^. Samples that were positive for hormone receptors (estrogen and progesterone) were those in which > 1% of the tumor cells showed positive nuclear staining of moderate to strong intensity on immunohistochemistry and were classified as Luminal A and B neoplasia if they presented the Ki67 index below or above 14%, respectively. Samples that exhibited > 10% of invasive tumor cells with strong HER2 staining in the plasma membrane were considered HER2 positive. In case of moderate staining in > 10% of the cells or strong staining in < 10% of the cells, the sample was re-evaluated by in situ hybridization and was considered positive if a HER2/centromere ratio > 2.0; or if a HER2/centromere ratio < 2.0 with mean HER2 > 6 signals per cell (greater than 120 signals in 20 nuclei). Tumor samples that did not express either hormonal or HER2 receptors were classified as TN BC^34^.

For sample calculation, the number of new cases of BC included for treatment at Hospital Pérola Byington during 2018 (2,985 cases) was taken into account; also the study design, with the comparison of outcome variables between two main groups (CTR vs. BC); the main outcome variables; the effect size of the variables according to the main studies published in the area; and the probability of committing a type 1 error (0.05) and type 2 error (0.20), with 80% power, resulted in 144 patients in each group (pairing 1:1).

### Blood collection

Venous blood was drawn after 12 h fasting and plasma was immediately isolated after centrifugation (3,000 rpm, 4 °C, 15 min). Plasma TC, TG, and HDLc were determined by enzymatic techniques. Low-density lipoprotein cholesterol (LDLc) was determined by the Friedewald formula^35^, and VLDLc as TG/5. ApoB was quantified by immunoturbidimetry (Randox Lab. Ltd. Crumlin, UK).

### Isolation of plasma lipoproteins

High-density lipoproteins (HDL; D = 1.063–1.21 g/mL) were isolated from BC and CTR women´s plasma by discontinuous density ultracentrifugation and immediately frozen at − 80 °C in a 5% saccharose solution. HDL composition in lipids (TC, TG, and PL) was determined by enzymatic techniques. ApoA-I was determined by the immunoturbidimetric method (Randox Lab. Ltd. Crumlin, UK).

### Low-density lipoprotein isolation and acetylation

LDL (D = 1.019–1.063 g/mL) was obtained by sequential ultracentrifugation of plasma from healthy volunteers and was purified by discontinuous density ultracentrifugation. After protein quantification by the Lowry technique^36^, LDL was incubated with acetic anhydride as previously described^37^. Acetylated LDL was dialyzed and utilized to load macrophages with cholesterol.

### HDL-mediated cholesterol efflux from bone marrow-derived macrophages

The Institutional Animal Care and Research Advisory Committee (Universidade Nove de Julho # 7,070,120,821, 08/23/2021) approved the study according to the U.S. National Institutes of Health Guide for the Care and Use of Laboratory Animals. All the methods were performed in accordance with ARRIVE guidelines (Supplementary file S1). Thirty male and female C57BL/6 J mice, aged 2–48 weeks, were housed with free access to commercial chow (Nuvilab CR1, São Paulo, Brazil) and drinking water in a conventional animal facility at 22 ± 2 °C under a 12 h light/dark cycle. Intraperitoneal overdose of ketamine hydrochloride (Ketalar) (300 mg/kg of body weight) and xylazine hydrochloride (Rompun) (30 mg/kg of body weight) were used to euthanize the animals, in accordance with the norms of the National Council for the Control of Animal Experimentation (CONCEA) of the Ministry of Science, Technology and Innovation (MCTI). Undifferentiated bone marrow cells were obtained from C57BL/6 wild-type mouse´s tibia and femora, as previously described^38^. Cells were differentiated into macrophages by incubating with L929 cells-conditioned medium (ATCC, American Tissue Culture Collection) and plated in culture dishes for 5 days at 37 °C, under 5% (v/v) CO_2_. The medium was changed for a new one and after 6 days replaced by DMEM (low glucose, containing 1% penicillin/streptomycin and 10% heat-inactivated fetal calf serum). Macrophages were overloaded with acetylated LDL (50 µg/mL) and ^14^C-cholesterol (0.3 µCi/mL) for 48 h, and after washing incubated with DMEM containing fatty acid-free albumin for equilibration of intracellular cholesterol pools. Cells were incubated for 6 h with HDL (50 µg/mL) from BC or control women; control incubations were performed in the absence of HDL (basal efflux). After incubation, the radioactivity in the medium was determined as well as in the cell lipid extract. The cholesterol efflux was determined as ^14^C-cholesterol in the medium / ^14^C-cholesterol in the medium + ^14^C-cholesterol in cells × 100. Values from basal efflux were subtracted from those obtained after incubation with HDL, in order to express the specific ability of HDL in removing cell cholesterol.

### Oxysterols quantification in isolated HDL

Oxysterols (24-hydroxycholesterol, 25-hydroxycholesterol, and 27-hydroxycholesterol) were measured from 1 mL of extracted HDL as previously described^39,40^. Briefly, a mixture of 100 ng of oxysterol deuterium-labeled (7α-hydroxycholesterol-d7, 7β-hydroxycholesterol-d7, 25-hydroxycholesterol-d7, 27-hydroxycholesterol-d7) (Avanti Polar Lipids, Alabaster, USA) was added as internal standard. Alkaline saponification was done by adding 10 mL of ethanolic KOH (0.4 M) solution for 2 h, at room temperature with adjustment to pH 7 with phosphoric acid. Then 20 mL of chloroform and 6 mL of water were added to the sample. After strong shaking and centrifugation at 4 °C, the aqueous phase was eliminated and the organic phase evaporated. The lipid extract was dissolved in toluene (1 mL). Oxysterols were separated from cholesterol by solid-phase extraction (Sigma- Aldrich Supelclean LC-Si SPE Tubes SUPELCO, Bellefonte, USA). The sample (1 mL in toluene) was placed in the column previously conditioned with 2 mL of hexane, following washing with 1 mL of hexane. Sterols were eluted with 1.5% isopropanol in hexane (8 mL), and oxysterols were further eluted with 30% isopropanol in hexane (6 mL). Then, the solvent was evaporated and samples were derivatized (100 μL of pyridine and 100 μL of *N*, *O*-bis (trimethylsilyl) trifluoroacetamide with trimethylchlorosilane (BSTFA; Sigma- Aldrich, St. Louis, USA), for 1 h at 60 °C). One microliter of the derivatized sample (1 µL) was injected into a gas chromatograph coupled to a mass spectrometer (Shimadzu GCMS-QP2010, Kyoto, Japan) by an automatic injector and analyzed in selected ion monitoring. The separation was carried out on a Restek capillary column (100% dimethyl polysiloxane-RxiR -1 ms. Cat. #13,323), 30 m, internal diameter 0.25 mm, for 30 min, using helium as mobile phase, with a constant linear velocity of 44.1 cm/sec. The oven started at 240 °C with an increment of 5◦C/min, for 7 min up to 290 °C. The mass spectrometer operated in impact electron mode at an ionization voltage of 70 eV with the temperature of the ion source at 300 °C^40^. Comparisons from standard curves peak areas to internal standards were made in order to quantify the oxysterols, as previously described^41^.

### Statistical analysis

The Shapiro–Wilk test was used to analyze normality; parametric data were represented by the mean and standard deviation of the sample and compared by Student's t-test, with or without Welch correction, depending on the performance of Levene's Test regarding sample sphericity. When analyzing more than two samples, analysis of variance of non-repeated measures was used with Tukey or Games-Howell post-test, according to the homo or heteroscedasticity, respectively; with bootstrap when necessary. Covariate analyses were performed to adjust the outcome variables with Sidak's post-test. Non-parametric data were represented by the median, lower and upper quartiles, and compared with each other using the Mann–Whitney test for two samples, and when comparing more than two samples, the Kruskal–Wallis test was used. If normalization was necessary, variables were log-transformed. Frequencies were compared using the Chi-square test. A value of *P* < 0.05 was considered statistically significant. IBM® SPSS Statistics (version 27.0), GraphPad Prisma (version 5.04) for Windows, and Microsoft® Excel for Mac (version 16.52) software were used for data tabulation and analysis.

### Ethical approval and informed consent

The study was conducted according to the guidelines of the Declaration of Helsinki and approved by the Institutional Ethics Committee of the Universidade Nove de Julho (# 3.139.460; February 2019); Centro de Referência da Saúde da Mulher (Hospital Pérola Byington; #3.225.220; March 2019) and Hospital das Clínicas da Faculdade de Medicina da Universidade de São Paulo (#3.317.909; March 2019).

## Results

In the BC group, the median age and the frequency of postmenopausal status, dyslipidemia and hypertension were higher in comparison to the CTR, although BMI, overweight (BMI $\ge$ 25 kg/m^2^) and reported statin use were similar between groups. Among tumors, the frequency (%) of histological types was: ductal (87.7), lobular (7.4), mucinous (4.3), and metaplastic (0.6). As expected, a higher frequency of LA and LB tumors was observed. 70.8% of BC women were categorized in clinical stages I and II, and 29.2% in advanced stages of disease (stages III and IV) (Table 1). The postmenopausal status was similar among clinical stages (stage I = 66.7%; stage II = 59.6%; stage III = 74.2%; and stage IV = 68.8%; χ^2^ = 2.011; *P* = 0.570). The luminal types (LA and LB) accounted for the majority of tumors (68%), with most cases classified as stages I and II (70.8%). Women with TN tumors had the highest frequency of advanced disease, with 60% being classified as stages III and IV, while those with LA tumors had the lowest percentage of advanced disease (8.9%)  (Supplementary file S2).

**Table 1:** Age, anthropometric and clinical data of CTR and BC women.

	CTR	BC	*P*		
n	150	163			
Age (years)*	51 (38–59)	55 (49–63)	< 0.001		
BMI (kg/m^2^)*	28 (25–31)	27 (24–31)	0.644		

	n	%	n	%	
BMI $\ge$ 25 kg/m^2^	108	73.5	106	68.4	0.179
Premenopausal	75	50.7	56	34.4	< 0.001
Postmenopausal	73	49.3	107	65.6	< 0.001
Dyslipidemia	18	12	35	21.9	0.021
Statin use	9	6	19	11.8	0.074
Hypertension	33	22.4	61	37.9	0.003
Histological type of BC					
Ductal			143	87.7	
Lobular			12	7.4	
Mucinous			7	4.3	
Metaplastic			1	0.6	
Molecular type of BC					
LA			46	28.2	
LB			65	39.8	
HER2			26	16	
TN			26	16	
Clinical stage of disease					
I			57	35.4	
II			57	35.4	
III			31	19.3	
IV			16	9.9	

*Values in median and interquartile ranges 25–75%. CTR, control women; BC, breast cancer women; BMI, body mass index; LA, luminal A; LB, luminal B; TN, triple-negative. Comparisons were done by the χ^2^ test.

Plasma lipid profile adjusted per age and the ratios TC/apoB and TG/HDLc were similar between CTR and BC groups (Table 2). In isolated HDL, the concentrations of TC, TG, and PL were lower in the BC group, while apoA-I was similar in both groups. The 27HC content in HDL was lower in BC as compared to CTR even after adjustment for age, but the concentrations of 24HC and 25HC in HDL were similar between groups. Despite some alterations in composition, HDL particles from BC and CTR presented similar abilities in mediating macrophage cholesterol removal (Table 2).

**Table 2:** Plasma lipids, HDL composition, and HDL-mediated cholesterol efflux in women with BC and CTR.

	CTR	BC	*P*
n*	132	126	
Age (years)	48 (37–58)	55 (49–63)	< 0.001
BMI (kg/m^2^)	28 (25–31)	27 (24–31)	0.391
TC (mg/dL)	172 (151–199)	178 (155–206)	0.423
TG (mg/dL)	84 (59–122)	92 (68–116)	0.224
apoB (mg/dL)	111 (89–139)	107 (82–141)	0.428
HDLc (mg/dL)	41 (35–51)	40 (32–48)	0.109
LDLc (mg/dL)	111 (95–131)	115 (95–142)	0.522
VLDLc (mg/dL)	17 (12–24)	18 (14–23)	0.224
non-HDLc (mg/dL)	127 (109–156)	136 (113–163)	0.252
TC/apoB (mg/dL)	1,5 (1,3–1,9)	1,7 (1,4–2,0)	0.065
TG/HDL (mg/dL)	1,9 (1,3–3,1)	2,3 (1,5–3,5)	0.091
HDL-TC (mg/dL)	44 (34–55)	37 (30–46)	0.001
HDL-TG (mg/dL)	14 (10–19)	13 (10–16)	0.039
HDL-PL (mg/dL)	98 (80–131)	87 (73–116)	0.006
HDL-apoA-I (mg/dL)	104 (82–141)	112 (82–142)	0.700
24HC (ng/mL)	38.9 (25.9–99.0)	34.9 (24.1–65.4)	0.203
25HC (ng/mL)	8.1 (5.1–10.7)	6.9 (5.4–9.3)	0.111
27HC (ng/mL)	12.6 (7.4–19.4)	7.7 (4.6–13.1)	0.002
^14^C-cholesterol efflux (%)	12 (9–18)	13 (9–19)	0.501

Plasma lipids were determined by enzymatic colorimetric methods and apoB by immunoturbidimetry. HDLc was determined after precipitation of lipoproteins containing apoB; non-HDLc was calculated as TC-HDLc. Oxysterols were quantified in isolated HDL by gas chromatography-mass spectrometry (GC–MS). The cholesterol efflux was determined in BMDM overloaded with ^14^C-cholesterol and acetylated LDL, utilizing 50 µg/mL of HDL as a cholesterol acceptor.

BMI, body mass index; TC, total cholesterol; TG, triglycerides; apoB, apolipoprotein B; HDLc, high-density lipoprotein cholesterol; LDLc, low density lipoprotein cholesterol; VLDLc, very low density lipoprotein cholesterol; PL, phospholipids; apoA-I, apolipoprotein A-I; 24HC, 24-hydroxycholesterol (CTR, n = 82; BC, n = 82); 25HC, 25-hydroxycholesterol (CTR, n = 82; BC, n = 82); 27HC, 27-hydroxycholesterol (CTR, n = 81; BC, n = 80). Comparisons were made using the Mann–Whitney test; data shown in median and interquartile ranges (25–75%). *P* values were age-adjusted.

When comparing the molecular types of BC, age and BMI were similar, but TC was higher in TN as compared to LA, LB, and HER2 tumors. Besides, plasma levels of TG, apoB, and non-HDLc were greater in TN as compared to LB and HER2 (Table 3). Although plasma lipids were different in TN tumors, the ratios TC/apoB (*P* = 0.065) and TG/HDLc (*P* = 0.091) did not reach statistical difference. Oxysterols in HDL were similar among LA, LB, HER2, and TN as well as the ability of cell cholesterol removal (Table 3).

**Table 3:** Plasma lipids, HDL composition, and HDL-mediated cholesterol efflux according to the molecular type of BC.

	LA	LB	HER2	TN	*P*
n	46	65	26	26	
Age (years)	60 (52–64)	54 (48–63)	55 (49–61)	53 (41–61)	0.107
BMI (kg/m^2^)	27 (24–31)	28 (25–31)	27 (24–31)	27 (24–30)	0.793
TC (mg/dL)	193 (162–228)	173 (155–205)	173 (151–193)	198 (173–214)	0.022^#^
TG (mg/dL)	103 (66–139)	84 (67–110)	82 (63–105)	102 (91–134)	0.026^$^
apoB (mg/dL)	129 (90–151)	98 (81–123)	100 (81–127)	134 (102–158)	0.005^$^
HDLc (mg/dL)	41 (34–49)	42 (33–53)	42 (32–52)	42 (36–50)	0.997
LDLc (mg/dL)	123 (99––155)	111 (90–138)	117 (85–134)	127 (98–148)	0.116
VLDLc (mg/dL)	21 (13–28)	17 (13–22)	16 (13–21)	20 (18–27)	0.026^$^
non-HDLc (mg/dL)	147 (121–184)	129 (104–162)	135 (105–151)	155 (130–174)	0.013^$^
TC/apoB	1.7 (1.4–2.0)	1.8 (1.5–2.1)	1.8 (1.5–2.0)	1.5 (1.3–2.0)	0.313
TG/HDLc	2.3 (1.3–3.4)	1.9 (1.3–3.1)	1.7 (1.3–3.2)	2.5 (2.0–3.4)	0.358
HDL-TC (mg/dL)	39 (30–49)	40 (27–52)	41 (29–49)	45 (23–58)	0.886
HDL-TG (mg/dL)	15 (12–34)	17 (11–62)	13 (11–18)	24 (14–91)	0.055
HDL-PL (mg/dL)	90 (71–119)	90 (77–121)	104 (82–124)	96 (77–117)	0.435
HDL-apoA-I (mg/dL)	114 (83–142)	108 (76–150)	108 (86–139)	107 (90–123)	0.894
24HC (ng/mL)	28 (22–49)	36 (23–78)	35 (28–58)	35 (24–88)	0.681
25HC (ng/mL)	7.3 (5.6–9.2)	8.3 (6.4–9.8)	6.6 (4.6–9.4)	6.0 (4.5–7.6)	0.110
27HC (ng/mL)	5.8 (4.5–10.6)	10.9 (4.4–13.8)	7.2 (4.7–14.6)	5.7 (4.1–14.2)	0.580
^14^C-cholesterol efflux (%)	14 (10–17)	12 (9–19)	13 (8–15)	11 (8–18)	0.921

Plasma lipids were determined by enzymatic colorimetric methods and apoB by immunoturbidimetry. HDLc was determined after precipitation of lipoproteins containing apoB; non-HDLc was calculated as TC-HDLc. Oxysterols were quantified in isolated HDL by gas chromatography-mass spectrometry (GC–MS). The cholesterol efflux was determined in BMDM overloaded with ^14^C-cholesterol and acetylated LDL, utilizing 50 µg/mL of HDL as a cholesterol acceptor.

CTR, control women; BC, breast cancer; BMI, body mass index; TC, total cholesterol; TG, triglycerides; apoB, apolipoprotein B; HDLc, high-density lipoprotein cholesterol; LDLc, low density lipoprotein cholesterol; VLDLc, very low density lipoprotein cholesterol; PL, phospholipids; apoA-I, apolipoprotein A-I; 24HC, 24-hydroxycholesterol (CTR, n = 82; BC, n = 82); 25HC, 25-hydroxycholesterol (CTR, n = 82; BC, n = 82); 27HC, 27-hydroxycholesterol (CTR, n = 81; BC, n = 80). Comparisons were made using the Kruskall-Wallis test with a significance of 0.05. Data shown in median and interquartile ranges (25–75%). LA, luminal A; LB, luminal B; TN, triple negative. ^#^ in comparison to LA, LB, and HER2; ^$^ in comparison to LB and HER2.

When subjects were categorized according to the stage of the disease reflected by increased levels of Ki67, it was observed that plasma lipids and their ratios were similar among groups. The composition of HDL in TC, PL, apoA-I, and oxysterols was similar among stages. Only HDL-TG was increased on stage III as compared to I, II, and IV. Reduced cholesterol efflux was observed with HDL isolated from BC women in more advanced stages of the disease (III and IV) as compared to stages I and II. (Table 4).

**Table 4:** Plasma lipids, HDL composition, and HDL-mediated cholesterol efflux according to clinical stages of BC.

	I	II	III	IV	*P*
n	57	57	31	16	
Age (years)	60 (49–64)	53 (46–61)	56 (51–61)	55 (48–66)	0.287
BMI (kg/m^2^)	27 (24–31)	28 (25–31)	27 (25–31)	25 (23–30)	0.291
Ki67	20 (10–30)	20 (10–30)	30 (20–70)	35 (30–72)	< 0.001 I–III < 0.001 I–IV < 0.001 II–III 0.015 II–IV 0.005 III–IV 0.416 I–II 0.122
TC (mg/dL)	187 (162–218)	179 (155–203)	189 (167–208)	183 (154–241)	0.600
TG (mg/dL)	94 (64–117)	92 (73–122)	92 (73–115)	84 (60–111)	0.760
apoB (mg/dL)	105 (81–138)	107 (87–137)	109 (84–141)	105 (84–150)	0.972
HDLc (mg/dL)	42 (33–51)	42 (33–50)	46 (39–55)	41 (33–45)	0.376
LDLc (mg/dL)	121 (98–146)	109 (92–137)	118 (97–139)	117 (98–172)	0.476
VLDLc (mg/dL)	21 (13–28)	17 (13–22)	16 (13–21)	20 (18–27)	0.760
non-HDLc (mg/dL)	140 (118–169)	132 (105–159)	139 (116–163)	132 (108–196)	0.640
TC/apoB	1.8 (1.4–2.1)	1.7 (1.4–2.0)	1.7 (1.4–2.0)	1.6 (1.2–2.0)	0.685
TG/HDLc	1.9 (1.5–3.2)	2.3 (1.4–3.3)	2.1 (1.0–3.3)	2.4 (1.6–2.7)	0.875
HDL-TC (mg/dL)	44 (30–51)	38 (28–49)	45 (26–67)	38 (31–51)	0.336
HDL-TG (mg/dL)	15 (12–44)	15 (11–39)	38 (14–92)	18 (10–56)	0.032 I–IV 0.831 II–IV 0.809 III–IV 0.035 I–II 0.968 –III 0.009 II–III 0.009
HDL-PL (mg/dL)	90 (75–119)	89 (76–114)	117 (87–124)	87 (79–113)	0.116
HDL-apoA-I (mg/dL)	116 (83–144)	104 (78–135)	114 (85–141)	109 (94–149)	0.733
24HC (ng/mL)	31 (19–48)	35 (26–75)	28 (25–50)	56 (34–128)	0.276
25HC (ng/mL)	6.5 (5.1–8.5)	7.1 (5.3–8.7)	9.7 (6.6–10.6)	6.0 (4.3–8.9)	0.247
27HC (ng/mL)	8.8 (4.6–15.1)	9.7 (4.6–14.4)	5.0 (3.9–9.1)	5.7 (3.4–10.1)	0.267
^14^C-cholesterol efflux (%)	14 (10–20)	13 (10–18)	9 (7–15)	9 (7–12)	0.011 III–IV 0.424 II–IV 0.014 I–IV 0.004 II–III 0.088 I–III 0.033 I–II 0.578

Plasma lipids were determined by enzymatic colorimetric methods and apoB by immunoturbidimetry. HDLc was determined after precipitation of lipoproteins containing apoB; non-HDLc was calculated as TC-HDLc. Oxysterols were quantified in isolated HDL by gas chromatography-mass spectrometry (GC–MS). The cholesterol efflux was determined in BMDM overloaded with ^14^C-cholesterol and acetylated LDL, utilizing 50 µg/mL of HDL as a cholesterol acceptor.

CTR, control women; BC, breast cancer; BMI, body mass index; TC, total cholesterol; TG, triglycerides; apoB, apolipoprotein B; HDLc, high-density lipoprotein cholesterol; LDLc, low density lipoprotein cholesterol; VLDLc, very low density lipoprotein cholesterol; PL, phospholipids; apoA-I, apolipoprotein A-I; 24HC, 24-hydroxycholesterol (CTR, n = 82; BC, n = 82); 25HC, 25-hydroxycholesterol (CTR, n = 82; BC, n = 82); 27HC, 27-hydroxycholesterol (CTR, n = 81; BC, n = 80). Comparisons were made using the Kruskall-Wallis test with a significance of 0.05. Data shown in median and interquartile ranges (25–75%).

## Discussion

The present study investigated plasma lipid levels, HDL composition, and functionality in newly diagnosed women with BC compared to CTR women, taking into account the molecular classification of the tumor and the clinical stage of the disease. The results showed that (1) BC cases (including all molecular types) had similar concentrations of plasma lipids adjusted for age but HDL particles less enriched in TC, PL, TG, and 27HC compared to CTR; (2) TN cases had a higher concentration of plasma lipids as compared to other molecular types of tumor; (3) HDL functionality along the first step of reverse cholesterol transport was impaired in advanced stages of BC, meaning less cholesterol removal from macrophages.

Plasma lipids are considered contributing factors for many types of cancer, including BC^42–46^, although the interpretation and comparison of studies are limited by the heterogeneity of BC, disease duration and staging, ethnic diversity, age, menopausal status, lifestyle, and oncological treatments, which are confounding factors. Furthermore, diabetes mellitus, insulin resistance, and obesity, which can alter lipoprotein profiles, are also present in many cases of BC as potential contributors to the disease development and progression. The current study exclusively included newly diagnosed, treatment-naïve participants, excluding smoking habit and women with diabetes mellitus and comorbidities that affect lipids. Moreover, the data were adjusted for age, which was higher in the BC group. As a result, many factors contributing to dyslipidemia were minimized, and the strict matching between the CTR and BC groups likely accounted for the absence of variation in the plasma lipid profile between all BC cases and CTR. Even with the exclusion data of women informing dylipidemia and/or statin use, results were similar (data not shown).

In the casuistic of the present investigation, as anticipated, the majority of tumors were of the luminal types in in stages I and II. Triple-negative cases had the highest frequency of advanced disease and higher levels of TC, apoB, nonHDLc, VLDLc, and TG compared to LA, LB, and HER-2. However, HDLc, composition, and functionality of the HDL particle were similar among all types. These alterations were independent of BMI and may represent a distinct characteristic of TN tumors, directing lipids as an energy source and structural components for tumor growth in a histotype with a more aggressive clinical course, lower survival rate, and a higher metastasis rate. These factors should be considered when exploring the association between plasma lipids and BC and deserves further investigation.

Also, findings are consistent with previous studies that have reported increased levels of VLDL-TG in TN BC. However, changes in CT/apoB and TG/HDLc did not reach statistical significance. A retrospective cohort study demonstrated that a high pretreatment TG/HDLc ratio was an independent predictor of the overall survival rate in TN tumors^47^. The enhanced expression of the LDL receptor and LDLR-related proteins 5 and 6 were found in TN tumors and associated with the higher ability of tumor growth and invasion; while the knockdown of LDLR-5 and 6 decreased tumorigenesis^48–51^.

A syngeneic tumor graft model demonstrated that tumor growth leads to changes in host lipid metabolism, promoting the synthesis of very low-density lipoproteins while inhibiting their metabolism, ultimately providing more energy to the tumor^52^. Furthermore, a targeted plasma liquid chromatography-tandem mass spectrometry (LC–MS/MS) approach identified potential lipid biomarkers in TN BC cases, such as ceramides, phosphatidylcholine, lysophosphatidylcholine, and diacylglycerol, thus confirming the alteration of the lipid profile in this molecular subtype^53^.

Unlike other studies, it was not found in the present investigation any changes in plasma lipids in cases of HER2-positive BC^45,54,55^. However, in individuals with HER2-positive BC, the analysis of the lipoprotein profile using nuclear magnetic resonance demonstrated an enhanced level of specific VLDL subfractions, which served as a marker of plasma lipid alteration compared to control women, even with similar BMI between the groups. Reductions in HDL subfractions were surrogate markers for the response to neoadjuvant chemotherapy follow-up^56^.

Although the ability of HDL to remove cell cholesterol was preserved in all women with BC when compared to CTR women, the reduced content of TC, PL, and 27HC in the HDL particles from BC cases may be a consequence of alterations in the tumor cells that hinder sterol exportation to HDL. In addition to the intrinsic ability of HDL to receive cholesterol, it is important to consider that lipid efflux is modulated by cellular components, such as the bioavailability of free or unesterified cholesterol and the content and functionality of HDL receptors sch as ABCA-1, ABCG-1, and SR-B1^57^. ABCA-1, which is expressed in the mammary gland, is indicated to be reduced in BC and associated with positive lymph nodes^58^. Its expression negatively affects the therapeutic efficiency of chemotherapy^59^ and is considered by some authors as a marker of TN tumors^60^. Conversely, its deficiency contributes to an increase in cellular and mitochondrial cholesterol content, which hinders cell death processes mediated by this organelle, thereby favoring tumor cell survival^24^.

Solid tumors are known to accumulate large amounts of cholesterol^48,61,62^, which is mainly due to the increased synthesis and uptake of lipoproteins^48,63^. The scavenger receptor class B type 1 (SR-B1) mediates the efflux of free cholesterol from cells and promotes its transfer to HDL. However, SR-B1 can also facilitate the uptake of modified lipoproteins, leading to the supply of cholesterol to cells and tumor progression^64^. Higher expression of SR-B1 is associated with increased aggressiveness and a worse prognosis of tumors^23,65,66^, while mutations in the *Scarb1* gene have been shown to inhibit tumor proliferation^67^.

Moreover, the reduced lipid content in HDL could be associated with the decreased detachment of surface components of TG-rich lipoproteins during lipolysis mediated by lipoprotein lipase, which is now recognized as a process of reverse transport of remnant cholesterol^68^. However, it is difficult to determine which mechanisms are involved in shaping the composition of HDL based on the present findings. These events cannot be captured by a simple determination of HDLc in plasma, which may explain some of the controversies surrounding the association between HDLc and BC.

According to the clinical stage of the disease, a diminished intrinsic capacity of the HDL particle to remove cell cholesterol was observed in stages III and IV in comparison to stages I and II. This observation was independent of changes in HDL composition and plasma lipids, highlighting the dissociation between plasma levels of HDLc and the overall functionality of HDL as a particle. Increasing levels of intracellular cholesterol promote the formation of oxysterols, which suggests an increase in the concentration of 27HC in the tumor microenvironment that negatively influences BC progression. The underlying mechanisms of the reduced HDL ability in removing cell cholesterol in advanced stages of BC were not investigated in the present study. HDL functionality may be impaired by its chemical modification resulting from glycoxidative and inflammatory stress that often accompanies cancer^69^. Consequently, a vicious circle may be established, which compromises tumor cholesterol homeostasis, exacerbating cancer progression and impairing HDL function. In this sense, HDL may even promote tumor growth, as experimental studies have previously shown that modified HDL was oncogenic^42,69^. Once again, these findings support the idea that the tumor's energy demand adapts systemic metabolism to its advantage.

The concept of reverse causation suggests that the tumor may modulate HDL, which may disassociate HDL from being a predictor of tumor risk. Therefore, HDL would serve more as a marker of tumor evolution rather than a protector or inducer of tumor genesis. Bone marrow-derived macrophages were used in this investigation to minimize cellular effects on HDL functionality measurement. However, it is crucial to consider that in vivo, the interplay between lipoprotein subsets and tumor cell biology determines HDL functionality and cell cholesterol content. Additional experiments employing BC cell lineages are necessary to enhance our comprehension of the interplay between plasma lipids, HDL, and BC.

To our knowledge, this is the first study demonstrating the loss of HDL function along the first step of reverse cholesterol transport according to disease burden, independent of plasma lipids. However, the study has certain limitations, such as the absence of detailed clinical data, including components of metabolic syndrome and visceral adiposity, as well as lifestyle information. Nevertheless, BMI was similar between the groups, and comorbidities that were associated with changes in lipid metabolism were excluded. Moreover, it was not identified the specific HDL structure components that were responsible for the impairment of cholesterol efflux, independently of lipids and apo A-I. A more detailed analysis of HDL proteomics, lipidomics, and microRNAs may provide additional information. A subanalysis of TN cases from this casuistic (n = 27) showed an enhanced antioxidant role of the HDL particle—reflected by retardation in LDL oxidation—that was positively associated to the apoA-I but independent of HDLc^70^.

The results of this investigation confirmed that TN tumors have increased levels of plasma lipids, potentially driving the more aggressive type of BC. This study also contributes to a better understanding of the role of HDL in BC, particularly in patients with a worse prognosis, where HDL's ability to remove excess cellular cholesterol is impaired. In comparison to the CTR group, only the composition of HDL was distinctive in subjects with BC. A prospective cohort study will aid in determining the prognostic value of HDL composition and functionality in predicting BC outcomes.

Finally, results contribute to the growing understanding of HDL's role in breast cancer pathophysiology. Since routine laboratory metrics cannot directly indicate this, it is essential to recognize the impact of HDL functionality on BC and develop new metrics accordingly.

## Supplementary Information

Supplementary Information 1.

Supplementary Information 2.

## Data Availability

All data reported are included in the manuscript and raw data can be kindly shared upon personal request to the corresponding author MP (m.passarelli@uni9.pro.br).
