# Supplementary material for: Increased plasma lipids in triple-negative breast cancer and impairment in HDL functionality in advanced stages of tumors
Source: Sci Rep. 2023 Jun 2;13:8998. doi: 10.1038/s41598-023-35764-7 (PMC10238519; doi:10.1038/s41598-023-35764-7)
Supplement: Supplementary file 2 — Supplementary Information 2. [file 41598_2023_35764_MOESM2_ESM.docx]

**S2 File. Crosstabulation: BC Molecular Types *versus* Disease Stages**

|  |  |  | **Stage I** | **Stage II** | **Stage III** | **Stage IV** | **Total** |
| --- | --- | --- | --- | --- | --- | --- | --- |
| **4 Molecular types** | **Luminal A** | Count (n) | 25 | 16 | 3 | 1 | 45 |
|  |  | % within 4 Molecular types | 55.6% | 35.6% | 6.7% | 2.2% | 100.0% |
|  |  | % within Clinical stage | 43.9% | 28.1% | 9.7% | 6.3% | 28.0% |
|  |  | % of Total | 15.5% | 9.9% | 1.9% | 0.6% | 28.0% |
|  | **Luminal B** | Count (n) | 21 | 26 | 10 | 8 | 65 |
|  |  | % within 4 Molecular types | 32.3% | 40.0% | 15.4% | 12.3% | 100.0% |
|  |  | % within Clinical stage | 36.8% | 45.6% | 32.3% | 50.0% | 40.4% |
|  |  | % of Total | 13.0% | 16.1% | 6.2% | 5.0% | 40.4% |
|  | **HER2** | Count (n) | 5 | 11 | 9 | 1 | 26 |
|  |  | % within 4 Molecular types | 19.2% | 42.3% | 34.6% | 3.8% | 100.0% |
|  |  | % within Clinical stage | 8.8% | 19.3% | 29.0% | 6.3% | 16.1% |
|  |  | % of Total | 3.1% | 6.8% | 5.6% | 0.6% | 16.1% |
|  | **Triple negative** | Count (n) | 6 | 4 | 9 | 6 | 25 |
|  |  | % within 4 Molecular types | 24.0% | 16.0% | 36.0% | 24.0% | 100.0% |
|  |  | % within Clinical stage | 10.5% | 7.0% | 29.0% | 37.5% | 15.5% |
|  |  | % of Total | 3.7% | 2.5% | 5.6% | 3.7% | 15.5% |
| **Total** |  | Count (n) | 57 | 57 | 31 | 16 | 161 |
|  |  | % within 4 Molecular types | 35.4% | 35.4% | 19.3% | 9.9% | 100.0% |
|  |  | % within Clinical stage | 100.0% | 100.0% | 100.0% | 100.0% | 100.0% |
|  |  | % of Total | 35.4% | 35.4% | 19.3% | 9.9% | 100.0% |

BC molecular types and disease stages values represented in absolute (n) and relative frequencies (%). LA =luminal A; LB = luminal B; TN= triple-negative. Comparisons were

done by the χ^2^ test (χ^2^= 31.618; P<0.001).
